# The *MLH1* c.1852_1853delinsGC (p.K618A) Variant in Colorectal Cancer: Genetic Association Study in 18,723 Individuals

**DOI:** 10.1371/journal.pone.0095022

**Published:** 2014-04-17

**Authors:** Anna Abulí, Luis Bujanda, Jenifer Muñoz, Stephan Buch, Clemens Schafmayer, Maria Valeria Maiorana, Silvia Veneroni, Tom van Wezel, Tao Liu, Helga Westers, Clara Esteban-Jurado, Teresa Ocaña, Josep M. Piqué, Montserrat Andreu, Rodrigo Jover, Angel Carracedo, Rosa M. Xicola, Xavier Llor, Antoni Castells, The EPICOLON Consortium, Malcolm Dunlop, Robert Hofstra, Annika Lindblom, Juul Wijnen, Paolo Peterlongo, Jochen Hampe, Clara Ruiz-Ponte, Sergi Castellví-Bel

**Affiliations:** 1 Department of Gastroenterology, Hospital Clínic, Centro de Investigación Biomédica en Red de Enfermedades Hepáticas y Digestivas (CIBEREHD), Institut d'Investigacions Biomèdiques August Pi i Sunyer (IDIBAPS), University of Barcelona, Barcelona, Catalonia, Spain; 2 Department of Gastroenterology, Hospital del Mar-IMIM (Hospital del Mar Medical Research Centre), Pompeu Fabra University, Barcelona, Catalonia, Spain; 3 Gastroenterology Department, Hospital Donostia, Networked Biomedical Research Centre for Hepatic and Digestive Diseases (CIBEREHD), Basque Country University, San Sebastián, Spain; 4 Department of Medine I, University Hospital Dresden, Dresden, Germany; 5 Department of General and Thoracic Surgery, University Hospital Schleswig-Holstein, Kiel, Germany; 6 IFOM, Fondazione Istituto FIRC di Oncologia Molecolare, Milan, Italy; 7 Department of Experimental Oncology and Molecular Medicine, Fondazione IRCCS Istituto Nazionale dei Tumori, Milan, Italy; 8 Department of Pathology, Leiden University Medical Center, Leiden, The Netherlands; 9 Department of Molecular Medicine and Surgery, Karolinska Institute, Stockholm, Sweden; 10 Department of Genetics, University Medical Center Groningen, Groningen, The Netherlands; 11 Department of Gastroenterology, Hospital General d'Alacant, Alicante, Spain; 12 Galician Public Foundation of Genomic Medicine (FPGMX), Centro de Investigación Biomédica en Red de Enfermedades Raras (CIBERER), Genomics Medicine Group, Hospital Clínico, Santiago de Compostela, University of Santiago de Compostela, Galicia, Spain; 13 Center of Excellence in Genomic Medicine Research, King Abdulaziz University, Jeddah, Kingdom of Saudi Arabia; 14 Section of Digestive Diseases and Nutrition, University of Illinois at Chicago, Chicago, Illinois, United States of America; 15 Colon Cancer Genetics Group, Institute of Genetics and Molecular Medicine, University of Edinburgh and MRC Human Genetics Unit, Edinburgh, United Kingdom; 16 Departments of Human Genetics and Clinical Genetics, Leiden University Medical Center, Leiden, The Netherlands; University of Illinois at Chicago, United States of America

## Abstract

Colorectal cancer is one of the most frequent neoplasms and an important cause of mortality in the developed world. Mendelian syndromes account for about 5% of the total burden of CRC, being Lynch syndrome and familial adenomatous polyposis the most common forms. Lynch syndrome tumors develop mainly as a consequence of defective DNA mismatch repair associated with germline mutations in *MLH1*, *MSH2*, *MSH6* and *PMS2*. A significant proportion of variants identified by screening these genes correspond to missense or noncoding changes without a clear pathogenic consequence, and they are designated as “variants of uncertain significance”, being the c.1852_1853delinsGC (p.K618A) variant in the *MLH1* gene a clear example. The implication of this variant as a low-penetrance risk variant for CRC was assessed in the present study by performing a case-control study within a large cohort from the COGENT consortium-COST Action BM1206 including 18,723 individuals (8,055 colorectal cancer cases and 10,668 controls) and a case-only genotype-phenotype correlation with several clinical and pathological characteristics restricted to the Epicolon cohort. Our results showed no involvement of this variant as a low-penetrance variant for colorectal cancer genetic susceptibility and no association with any clinical and pathological characteristics including family history for this neoplasm or Lynch syndrome.

## Introduction

Colorectal cancer (CRC) is one of the most frequent neoplasms and an important cause of mortality in the developed world. This cancer is caused by both genetic and environmental factors although 35% of the variation in CRC susceptibility involves inherited genetic differences. Mendelian syndromes account for about 5% of the total burden of CRC, being Lynch syndrome and familial adenomatous polyposis the most common forms. Lynch syndrome tumors develop mainly as a consequence of defective DNA mismatch repair (MMR) associated with germline mutations in the *MLH1*, *MSH2*, *MSH6* and *PMS2* genes [1]. Once clinical criteria for this syndrome are complied, genetic screening of these genes is performed when a MMR defect is detected in the patient's tumor. When a pathogenic variant is detected, management of this disease can be significantly improved by identifying carriers that will benefit from specific screening, preventive, and therapeutic measures. Also, identifying non-carriers in additional family members permit to release these individuals from intensive surveillance. Noteworthy, a significant proportion of variants identified in the MMR genetic screening correspond to missense or noncoding changes without a clear pathogenic consequence, and they are designated as “variants of uncertain significance” (VUS). Therefore, differentiating pathogenic and neutral genetic variants is still a major challenge in clinical genetics [2].

The c.1852_1853delinsGC (p.K618A) variant in the *MLH1* gene corresponds to a clear example of VUS in Lynch syndrome. When consulting the Leiden Open Variation Database (LOVD v.2.0), there are 120 entries for this variant [3]. Available past studies reached contradictory conclusions about its pathogenicity reporting harmful *in silico* predictions [4], absence of splicing or mRNA alteration [5], presence in patients with a defective MMR tumor [6], co-occurrence with clearly pathogenic MMR mutations [7], apparent segregation with disease [8], and a majority of non-altered *in vitro* functional studies [9], [10]. All previous data permitted to categorize it in LOVD as a class 1 variant (non-pathogenic/low clinical significance) [11]. Therefore, it should be considered as a neutral variant in terms of its implication with Lynch syndrome.

Recently, genome-wide association studies (GWAS) successfully identified so far 30 common, low-penetrance susceptibility variants in 25 risk loci for CRC [12]–[19]. Some genetic variants in hereditary CRC genes labeled as VUS could constitute low-penetrance risk alleles for CRC. Indeed, this hypothesis has been previously tested for some variants in those genes [20]. In agreement with this rationale, the main aim of the present study was to assess the implication of the c.1852_1853delinsGC (p.K618A) variant in the *MLH1* gene as a low-penetrance risk variant for CRC by performing a case-control study within a large cohort from the COGENT consortium-COST Action BM1206, an international effort to facilitate the study of inherited genetic predisposition to CRC [21], [22].

## Materials and Methods

### Study population

The current genetic association study totalized 8,055 CRC cases and 10,668 controls from 7 different cohorts (Edinburgh, Epicolon, Groningen, Kiel, Leiden, Milano, Stockholm) and recruitment details are summarized below. The study was approved by the institutional ethical committee of each participating hospital and written informed consent was obtained from all patients.

#### Edinburgh cohort (1,553 CRC cases and 932 controls)

A population-based series of patients from throughout Scotland, who were diagnosed with colorectal cancer when they were less than 55 years of age, were recruited to the study between February 1999 and June 2004. During the same period, unaffected controls were ascertained from a population-based register (community health index) and were invited to participate.

#### Epicolon (2,001 CRC cases and 1,647 controls)

Cases and controls were recruited through the EPICOLON Consortium that is based on a prospective, multicenter and population-based epidemiology survey of the incidence and features of CRC in the Spanish population [23]. Briefly, cases were selected as patients with *de novo* histologically confirmed diagnosis of colorectal adenocarcinoma. Exclusion criteria were hereditary CRC forms, such as Lynch syndrome and familial adenomatous polyposis (FAP) and a personal history of inflammatory bowel disease. Controls were from the Spanish National DNA bank and were confirmed not to have cancer or history of neoplasm and no family history of CRC. All cases and controls were of Caucasian ethnicity.

#### Groningen (559 CRC cases and 501 controls)

Unselected CRC cases and hospital patient controls from the Netherlands included in the SCOPE project.

#### Kiel (1,768 CRC cases and 2.030 controls)

Cases and controls from population-based biobank projects including POPGEN (Population Genetic Cohort) from Schleswig-Holstein, north Germany, and SHIP (Survey of Health in Pommerania) from east and north-east Germany.

#### Leiden (505 CRC cases and 836 controls)

Cases and controls were recruited as previously described [24]. Briefly, most of the cases were recruited through the clinical genetics department. All cases were diagnosed with CRC and had early onset and/or positive family history for CRC. Known dominant polyposis syndromes, HNPCC/Lynch syndrome or bi-allelic MutYH mutation carriers were excluded. A single proband from each family was included in this study. Controls were healthy blood donors from the southwest region of the Netherlands. All cases and controls were of Caucasian ethnicity.

#### Milano (619 CRC cases and 2,526 controls)

Briefly, the cases were consecutive individuals affected with CRC who underwent surgery at the Fondazione IRCCS Istituto Nazionale Tumori in Milan (INT). The controls were blood donors recruited through the Immunohematology and Transfusion Medicine Service of INT the Associazione Volontari Italiani Sangue Comunale in Milan. All cases and controls were of Caucasian ethnicity.

#### Stockholm (1,729 CRC cases and 1,487 controls)

Unselected cases ascertained through 12 hospitals serving the Stockholm-Gotland and Uppsala-Örebro health-care regions in Sweden and blood donor controls.

### Genotyping

**Figure 1:**
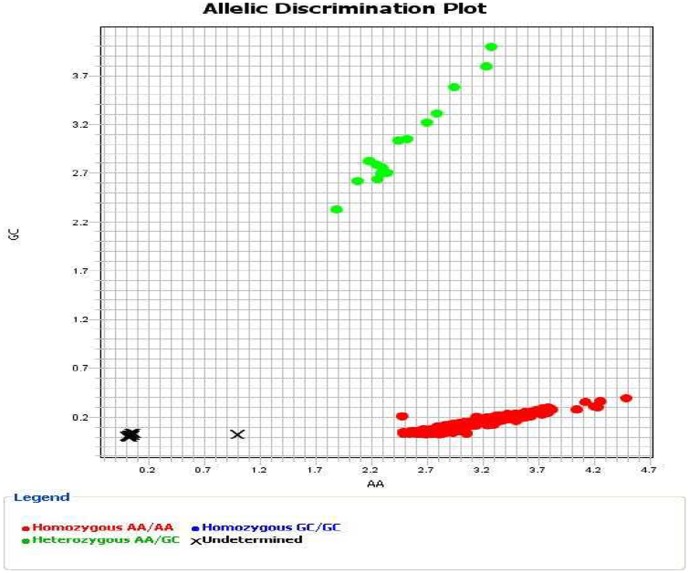
Allelic discrimination for c.1852_1853delinsGC (p.K618A) variant in the *MLH1* gene by using the TaqMan system. Red dots correspond to non-carriers (AA/AA genotype) and green dots to heterozygous carriers (AA/GC).

DNA was obtained from peripheral blood by standard extraction procedures. Allelic discrimination to genotype the c.1852_1853delinsGC (p.K618A) variant in the *MLH1* gene was performed by using a custom assay with the TaqMan allelic discrimination system (Life Technologies, Foster City, USA). As quality control, DNA from a known carrier of this variant was used as positive control, as well as duplicates and negative controls for amplification. Data could be available upon request. An example of allelic discrimination for this variant is shown in **Figure 1**.

### Statistical analysis

To test the association between the c.1852_1853delinsGC (p.K618A) variant in the *MLH1* gene and CRC risk, odds ratios (OR) and 95%CI were calculated for each genotype by using PLINK v1.07 [25], separately in each cohort and globally. No deviation of the genotype frequency in controls from those expected under Hardy-Weinberg equilibrium (HWE) was detected by χ^2^ test (1 df) (P-value = 0.6294) [26].

In order to explore if personal and/or familial characteristics were associated with the presence of the c.1852_1853delinsGC (p.K618A) variant in the *MLH1* gene, univariate analysis was performed restricted to the CRC cases from the Epicolon cohort due to data availability in this cohort. The selected clinical variables to be evaluated were gender, age (dichotomized by 50 y.o.), location of CRC, previous neoplasm, previous/synchronous adenoma, CRC familiy history (any relative with CRC), Lynch syndrome family history (any relative affected), microsatellite instability (MSI) and TNM tumor stage. Categorical variables were compared by the χ^2^ test (1 df), applying the Yates' correction when needed. All *P*-values were two-sided, and a value less than 0.05 was considered statistically significant. Calculations were performed using the SPSS software version 18.0 (SPSS Inc, Chicago, Ill).

## Results and Discussion

Genotyping for the c.1852_1853delinsGC (p.K618A) variant in the *MLH1* gene was successful in 8,055 CRC cases and 10,668 controls from 7 independent cohorts. Percentage of carriers varied between 0.4–2.6% in CRC cases and 0.5–3.1% for controls in the different cohorts, being 1.4% and 1.5% in the entire cohort for CRC cases and controls, respectively. Genotypic association results are shown in **Table 1** for each cohort and globally. No association of this variant with CRC risk was detected neither in a specific cohort nor globally.

**Table 1:** Genotypic association results for the *MLH1* c.1852_1853delinsGC (p.K618A) variant in 18,723 individuals from 7 cohorts.

Cohort	Controls	%	Cases	%	OR	lower	upper	*P*-value
*Edinburgh*								
AA/AA	1,539	99.1	916	98.3	1.000			0.087
AA/GC	14	0.9	16	1.7	1.904	0.934	3.884	
Total	1,553		932					
*Epicolon*								
AA/AA	1,596	96.9	1,949	97.4	1.000			0.368
AA/GC	51	3.1	52	2.6	0.839	0.574	1.228	
Total	1,647		2,001					
*Groningen*								
AA/AA	555	99.3	497	99.2	1.000			1.000
AA/GC	4	0.7	4	0.8	1.116	0.281	4.438	
Total	559		501					
*Kiel*								
AA/AA	2,003	98.7	1,752	99.1	1.000			0.282
AA/GC	27	1.3	16	0.9	0.680	0.368	1.259	
Total	2,030		1,768					
*Leiden*								
AA/AA	832	99.5	503	99.6	1.000			1.000
AA/GC	4	0.5	2	0.4	0.828	0.152	4.503	
Total	836		505					
*Milano*								
AA/AA	2,526	98.8	614	99.2	1.000			0.525
AA/GC	30	1.2	5	0.8	0.688	0.268	1.767	
Total	2,556		619					
*Stockholm*								
AA/AA	1,466	98.6	1,700	98.3	1.000			0.571
AA/GC	21	1.4	29	1.7	1.188	0.680	2.074	
Total	1,487		1,729					
***GLOBAL***								
AA/AA	10,517	98.6	7,931	98.5	1.000			0.501
AA/GC	151	1.4	124	1.5	1.088	0.859	1.377	
** Total**	**10,668**		**8,055**					

OR, odds ratio.

In order to further explore the putative implication of this *MLH1* variant with CRC risk, we performed a case-only genotype-phenotype correlation restricted to the Epicolon cohort (2,001 CRC cases) with several clinical and pathological characteristics. Results are shown in **Table 2**. Again, none of the analyzed variables showed a distinct association with the presence of the c.1852_1853delinsGC (p.K618A) variant. Results for CRC family history and Lynch syndrome family history were statistically significant but the presence of any of these variables was linked with the wild-type genotype (AA/AA). The rest of variables showed a similar distribution between carriers and non-carriers.

**Table 2:** Genotype-phenotype correlation of the *MLH1* c.1852_1853delinsGC (p.K618A) variant with clinical and pathological characteristics in colorectal cancer cases from the Epicolon cohort.

	CRC≤50	%	CRC>50	%	OR	lower	upper	*P*-value
**Age**								
AA/AA	97	5	1,841	95	1.000			1.000
AA/GC	2	3.8	50	96.2	1.317	0.316	5.493	
Total	99		1,891					
	Female	%	Male	%	OR	lower	upper	*P*-value
**Gender**								
AA/AA	766	39.5	1,172	60.5	1.000			0.388
AA/GC	17	32.7	35	67.3	1.346	0.749	2.419	
Total	783		1,207					
	Colon	%	Rectum	%	OR	lower	upper	*P*-value
**CRC location**								
AA/AA	1,267	65.9	656	34.1	1.000			0.882
AA/GC	33	64.7	18	35.3	1.053	0.589	1.885	
Total	1,300		674					
	No	%	Yes	%	OR	lower	upper	*P*-value
**Previous neoplasm**								
AA/AA	1,290	73.8	458	26.2	1.000			0.624
AA/GC	39	78	11	22	0.794	0.403	1.564	
Total	1,329		469					
	No	%	Yes	%	OR	lower	upper	*P*-value
**Prev/sync adenoma**								
AA/AA	1,268	71.2	513	28.8	1.000			0.112
AA/GC	41	82	9	18	0.543	0.262	1.124	
Total	1,309		522					
	No	%	Yes	%	OR	lower	upper	*P*-value
**CRC FH**								
AA/AA	1,652	85.2	286	14.8	1.000			0.026
AA/GC	50	96.2	2	3.8	0.231	0.056	0.955	
Total	1,702		288					
	No	%	Yes	%	OR	lower	upper	*P*-value
**Lynch FH**								
AA/AA	1,401	81.5	317	18.5	1.000			0.048
AA/GC	42	93.3	3	6.7	0.316	0.097	1.025	
Total	1,443		320					
	No	%	Yes	%	OR	lower	upper	*P*-value
**MSI**								
AA/AA	1,308	94	84	6	1.000			0.731
AA/GC	37	92.5	3	7.5	1.263	0.381	4.180	
Total	1,345		87					
	I–II	%	III–IV	%	OR	lower	upper	*P*-value
**TNM**								
AA/AA	909	53.7	783	46.3	1.000			1.000
AA/GC	26	53.1	23	46.9	1.027	0.581	1.814	
Total	935		806					

CRC, colorectal cancer; OR, odds ratio; Prev/Sync, Previous/Synchronous; FH, family history; MSI, microsatellite instability; TNM, tumor-node-metastasis.

Obviously, genetic variants causing a missense mutation have a less clear pathogenic interpretation than those causing a premature termination of the protein. The c.1852_1853delinsGC (p.K618A) variant in the *MLH1* gene is a prominent example of a VUS that has been controversial for many years in the context of Lynch syndrome genetic diagnosis. However, recent functional studies have permitted to characterize more thoroughly its real effect of the MLH1 protein and it can be concluded that its effect is neutral or with very subtle effect [5], [8]–[11].

Regarding its putative implication in CRC risk as a rare low-penetrance variant, previous studies were sparse and included a small number of CRC cases and controls [27], [28]. Consequently, it was justified to perform a case-control association study in a large cohort in order to reach more solid conclusions. Our results showed no involvement of this variant in CRC risk as a low-penetrance variant in the *MLH1* gene.

Regarding its putative implication in familial CRC, this variant was also seen to be over-represented in families with suspected Lynch syndrome in a previous study [29]. Our results will be not in agreement with this previous observation since the K618A variant was not linked in the Epicolon cohort to the presence of CRC family history and Lynch syndrome family history. Therefore, our study is adding to the existing literature by showing that this variant is not linked to familial CRC.

Finally, we can conclude from our results and previous evidence that the c.1852_1853delinsGC (p.K618A) variant in the *MLH1* gene should be regarded from now on as a polymorphism without functional effect on the MLH1 protein, no role in genetic predisposition to Lynch syndrome, as well as no apparent effect as a low-penetrance variant for CRC genetic susceptibility.

## Supporting Information

Appendix S1 **Members of the EPICOLON Consortium (Gastrointestinal Oncology Group of the Spanish Gastroenterological Association).**
(DOCX)
