# Supplementary material for: The MLH1 c.1852_1853delinsGC (p.K618A) Variant in Colorectal Cancer: Genetic Association Study in 18,723 Individuals
Source: PLoS One. 2014 Apr 17;9(4):e95022. doi: 10.1371/journal.pone.0095022 (PMC3990597; doi:10.1371/journal.pone.0095022)
Supplement: Appendix S1 — Members of the EPICOLON Consortium (Gastrointestinal Oncology Group of the Spanish Gastroenterological Association). (DOCX) [file pone.0095022.s001.docx]

Hospital 12 de Octubre, Madrid: Juan Diego Morillas (local coordinator), Raquel Muñoz, Marisa Manzano, Francisco Colina, Jose Díaz, Carolina Ibarrola, Guadalupe López, Alberto Ibáñez; Hospital Clínic, Barcelona: Antoni Castells (local coordinator), Virgínia Piñol, Sergi Castellví-Bel, Francesc Balaguer, Victoria Gonzalo, Teresa Ocaña, María Dolores Giráldez, Maria Pellisé, Anna Serradesanferm, Leticia Moreira, Miriam Cuatrecasas, Josep M. Piqué; Hospital Clínico Universitario, Zaragoza: Ángel Lanas (local coordinator), Javier Alcedo, Javier Ortego; Hospital Cristal-Piñor, Complexo Hospitalario de Ourense: Joaquin Cubiella (local coordinator), Mª Soledad Díez, Mercedes Salgado, Eloy Sánchez, Mariano Vega; Parc de Salut Mar, Barcelona: Montserrat Andreu (local coordinator), Anna Abuli, Xavier Bessa, Mar Iglesias, Agustín Seoane, Felipe Bory, Gemma Navarro, Beatriz Bellosillo; Josep Mª Dedeu, Cristina Álvarez, Marc Puigvehí; Hospital San Eloy, Baracaldo and Hospital Donostia, CIBERehd, University of Basque Country, San Sebastián: Luis Bujanda (local coordinator) Ángel Cosme, Inés Gil, Mikel Larzabal, Carlos Placer, María del Mar Ramírez, Elisabeth Hijona, Jose M. Enríquez-Navascués, Jose L. Elosegui; Hospital General Universitario de Alicante: Artemio Payá (EPICOLON I local coordinator), Rodrigo Jover (EPICOLON II local coordinator), Cristina Alenda, Laura Sempere, Nuria Acame, Estefanía Rojas, Lucía Pérez-Carbonell; Hospital General de Granollers: Joaquim Rigau (local coordinator), Ángel Serrano, Anna Giménez; Hospital General de Vic: Joan Saló (local coordinator), Eduard Batiste-Alentorn, Josefina Autonell, Ramon Barniol; Hospital General Universitario de Guadalajara and Fundación para la Formación e Investigación Sanitarias Murcia: Ana María García (local coordinator), Fernando Carballo, Antonio Bienvenido, Eduardo Sanz, Fernando González, Jaime Sánchez, Akiko Ono; Hospital General Universitario de Valencia: Mercedes Latorre (local coordinator), Enrique Medina, Jaime Cuquerella, Pilar Canelles, Miguel Martorell, José Ángel García, Francisco Quiles, Elisa Orti; CHUVI-Hospital Meixoeiro, Vigo: EPICOLON I: Juan Clofent (local coordinator), Jaime Seoane, Antoni Tardío, Eugenia Sanchez. EPICOLON II Mª Luisa de Castro (local coordinator), Antoni Tardío, Juan Clofent, Vicent Hernández; Hospital Universitari Germans Trias i Pujol, Badalona and Section of Digestive Diseases and Nutrition, University of Illinois at Chicago, IL, USA: Xavier Llor (local coordinator), Rosa M. Xicola, Marta Piñol, Mercè Rosinach, Anna Roca, Elisenda Pons, José M. Hernández, Miquel A. Gassull; Hospital Universitari Mútua de Terrassa: Fernando Fernández-Bañares (local coordinator), Josep M. Viver, Antonio Salas, Jorge Espinós, Montserrat Forné, Maria Esteve; Hospital Universitari Arnau de Vilanova, Lleida: Josep M. Reñé (local coordinator), Carmen Piñol, Juan Buenestado, Joan Viñas; Hospital Universitario de Canarias: Enrique Quintero (local coordinator), David Nicolás, Adolfo Parra, Antonio Martín; Hospital Universitario La Fe, Valencia: Lidia Argüello (local coordinator), Vicente Pons, Virginia Pertejo, Teresa Sala; Hospital Sant Pau, Barcelona: Dolors Gonzalez (local coordinator) Eva Roman, Teresa Ramon, Maria Poca, Mª Mar Concepción, Marta Martin, Lourdes Pétriz; Hospital Xeral Cies, Vigo: Daniel Martinez (local coordinator); Fundacion Publica Galega de Medicina Xenomica (FPGMX), CIBERER, Genomic Medicine Group-University of Santiago de Compostela, Santiago de Compostela, Galicia, Spain: Ángel Carracedo (local coordinator), Clara Ruiz-Ponte, Ceres Fernández-Rozadilla, Mª Magdalena Castro; Hospital Universitario Central de Asturias: Sabino Riestra (local coordinator), Luis Rodrigo; Hospital de Galdácano, Vizcaya: Javier Fernández (local coordinator), Jose Luis Cabriada; Fundación Hospital de Calahorra (La Rioja) La Rioja: Luis Carreño (local coordinator), Susana Oquiñena, Federico Bolado; Hospital Royo Villanova, Zaragoza: Elena Peña (local coordinator), José Manuel Blas, Gloria Ceña, Juan José Sebastián; Hospital Universitario Reina Sofía, Córdoba: Antonio Naranjo (local coordinator).
